# Methods for Analyzing Lipid Droplets in Mammalian Oocytes and Fertilized Embryos

**DOI:** 10.1002/rmb2.70002

**Published:** 2025-12-01

**Authors:** Megumi Ibayashi, Satoshi Tsukamoto

**Affiliations:** ^1^ Laboratory Animal and Bioresource Sciences Section National Institutes for Quantum Science and Technology Chiba Anagawa Japan

**Keywords:** embryo, lipid droplet, methods, mouse, oocyte

## Abstract

**Background:**

Lipid droplets (LDs) are organelles consisting of a central core of neutral lipids covered by a single layer of phospholipids and are found in most eukaryotic cells. It has long been known that mammalian oocytes accumulate different amounts of LDs between animals; however, it is largely unknown why LD content varies from animal to animal and its physiological role remains unclear. Reflecting the growing interest in LDs in mammalian oocyte and early embryos, several comprehensive reviews have appeared in the last few years, but none have reviewed methods for visualizing and analyzing LDs stored in oocytes or fertilized eggs.

**Methods:**

We outline experimental methods for visualizing LDs in mammalian oocytes and early embryos. We also describe a method for LD degradation by fertilization‐induced autophagy and a centrifugation‐based method for the removal of LDs from ovulated metaphase II (MII) oocytes in mice.

**Main Findings:**

This review outlines the advantages and disadvantages of some of the typical methods for observing and analyzing LDs in oocytes and fertilized embryos.

**Conclusion:**

Our review provides useful information not only to basic researchers interested in LDs in mammalian oocytes and fertilized embryos, including humans, but also to embryologists and medical doctors.

## 1 Introduction

Lipid droplets (LDs) are organelles that store neutral lipids such as triglycerides and cholesterol esters in a core surrounded by a phospholipid monolayer and play an important role in lipid metabolism [1, 2, 3]. In eukaryotes, LDs are found in most cells and tissues, but they vary in size and number. Even within the same cell, LDs are heterogeneous (e.g., the lipid composition within LDs and the proteins on their LD surface are different) [4, 5].

**FIGURE 1:**
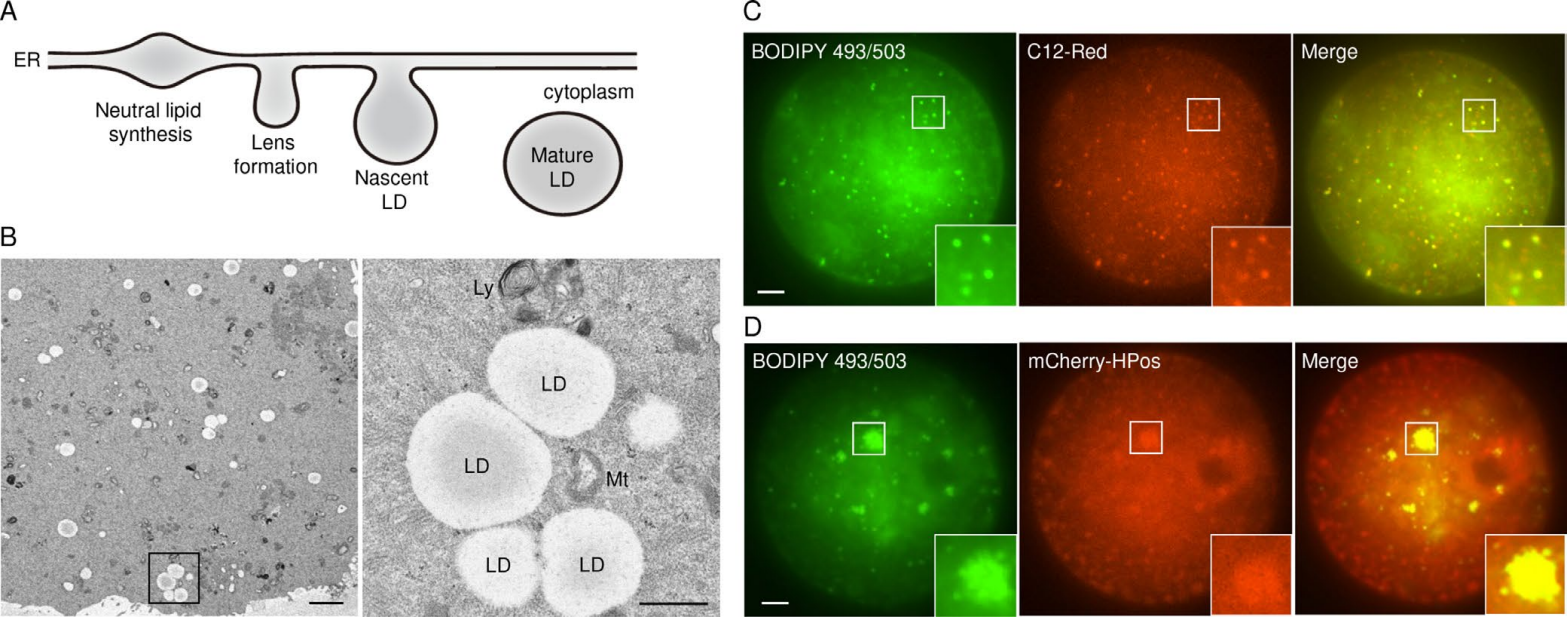
(A) Model of LD formation and expansion in the ER. (B) Electron microscope images showing mouse 2‐cell embryos. Right panel shows higher‐magnification images of the boxed area. Scale bars: 2 μm (left), 500 nm (left). LD, lipid droplet; Mt., mitochondria; Ly, lysosome. (C) Fluorescence microscopy image showing LDs in MII oocytes, which were stained with both BODIPY 493/503 (green) and C12‐Red (red). (D) MII‐oocyte microinjected with mCherry‐HPos mRNA were stained with BODIPY 493/503, and observed by laser confocal fluorescence microscopy. Insets show higher‐magnification images of the representative boxed areas. Scale bars: 20 μm.

The LD synthesis pathway roughly consists of four processes (Figure 1A). The first is neutral lipid synthesis in the endoplasmic reticulum (ER), which involves enzymes such as glycerol‐3‐phosphate acyltransferase (GPAT), diacylglycerol acyltransferase (DGAT), acyl‐CoA cholesterol O‐acyltransferases 1 and 2 (ACAT1 and ACAT2), and Sterol O‐acyltransferase 1 (SOAT1) [6, 7]. The second is the formation of lens‐like structures between ER membranes by the accumulation of neutral lipids, which is more likely to occur in the ER tubules than in the sheets [8]. The third is the detachment of LDs from the ER: when a sufficient amount of neutral lipids accumulate within the lens‐like structures, LDs bud into the cytoplasm. The fourth is the growth of LDs: within minutes to hours after LDs bud from the ER, they expand in size. The birthplace of LDs on the ER is determined by Berardinelli‐Seip Congenital Lipodystrophy (BSCL2, also known as seipin) and its functional partner lipid droplet assembly factor 1 (LDAF1) [9, 10]. Seipin supports the formation of structurally uniform ER‐LD contacts and promotes the transport of triglycerides from the ER to LDs. Furthermore, the seipin‐LDAF1 complex acts as machinery that determines the site of initial LD formation in the ER [10].

The surface of LDs contains unique proteins with diverse functions that can be classified into two groups: class I (also known as *ER to LD* [ERTOLD]) proteins, such as associated with LD protein 1 (ALDI) and adipose triglyceride lipase (ATGL), which are ER‐localized and translocate to the surface layer of LDs formed on the ER [11, 12, 13]; and class II (also known as *cy*tosol *to LD* [CYTOLD]) proteins, including perilipin (PLIN) proteins (PLIN1–5) [14], which are involved in the regulation of LD size, localize in the cytoplasm and translocate to the surface layer of mature LDs (see reviewed by Olarte et al.) [15]. To date, more than 150 LD‐associated proteins have been identified and are available in the Lipid Droplet Knowledge Portal (https://lipiddroplet.org), an international LD‐associated protein database [16]. These include proteins that are not involved in lipid metabolism at all, indicating that LDs not only act as a store of lipids, but also play a variety of roles.

LDs are degraded by two major pathways: lipolysis, in which the neutral lipids stored in LDs are hydrolyzed in a stepwise manner by lipases, such as ATGL and hormone‐sensitive lipase (HSL); and autophagic degradation (known as lipophagy), in which some LDs are engulfed by autophagosomes that fuse with lysosomes, resulting in their degradation by lysosomal acid lipases [17]. The resulting degradation products, i.e., free fatty acids and cholesterol, can be utilized as substrates for energy and hormone production within cells.

It is well‐known that mammalian oocytes and early embryos contain LDs. Interestingly, the content of LDs in oocytes and early embryos varies greatly among animal species [18]. For example, LDs are abundant in porcine and bovine oocytes and early embryos but are scarce in mice and humans. In pigs, LDs already exist in the oocytes of primordial follicles, whereas in mice, LDs emerge in the oocytes of secondary follicles [19, 20]. Further research is needed to clarify how this temporary difference in LD biosynthesis affects LD content in oocytes/embryos across animal species.

The morphology of LDs stored in oocytes changes drastically after fertilization. For example, in mice, LDs in MII oocytes before fertilization are aggregated (depending on the mouse strain), but after fertilization, they are dispersed throughout the cytoplasm. Then, after compaction, individual LDs fuse together to form one large LD in a process mediated by cell death‐inducing DFF45‐like effector A (CIDEA), which is involved in LD enlargement as an LD surface protein [21].

Why do LDs dramatically alter their morphology after fertilization? Early embryonic development immediately after fertilization is dependent on energy substrates such as pyruvate and sodium lactate, but from the 8‐cell stage onward, glucose is the main energy substrate (i.e., the glycolytic system is activated) [22]. The increase in LD size coinciding with elevated glycolytic activity suggests that LD morphology may change in response to energy metabolism during preimplantation embryonic development. Therefore, LD morphology is thought to reflect the energy level in the early embryo.

Ptak's group demonstrated that LDs can be a source of nutrients under delayed implantation [23]. Mammalian blastocysts undergo delayed implantation (i.e., become dormant without implantation) depending on maternal physiological conditions. Furthermore, through comprehensive lipidome analysis, which can be conducted on a small number of oocytes and fertilized embryos, Zhang's group revealed that large‐scale changes in lipid profiles (lipid remodeling) occur during preimplantation embryonic development [24]. This research has shown that lipid remodeling contributes to the establishment of polarity and blastocyst formation, which are necessary for development, particularly after the 8‐cell stage.

As such, a growing number of studies have focused on lipid metabolism linked to LDs in mammalian oocytes and fertilized embryos. This review outlines the advantages and disadvantages of some of the typical methods for observing LDs in oocytes and fertilized embryos (Table 1). In addition, we discuss methods for degrading or removing LDs in oocytes and fertilized embryos by autophagic degradation or two‐step centrifugation, respectively.

**TABLE 1:** Representative methods to visualize LDs in oocytes and early embryos are listed with their advantages and disadvantages. See text for details of each method.

Methods for visualizing LDs in oocytes/embryos	Advantages	Disadvantages	References
Electron microscopy	LD ultrastructure can be analyzed	Fixation required	Ibayashi et al. [25]
	No staining	Time consuming	Ohsaki et al. [26]
		Highly specialized equipment required	
Raman scattering microscopy	Lipid distribution can be imaged	Highly specialized equipment required	Bradley et al. [27]
	No staining	Weak signal	Le et al. [28]
	Can be used for live cell and fixed cell observation		
Fluorescence labeling: BODIPY 493/503, C12‐Red	Easy staining	Low photostability	Thumser et al. [29]
	Can be used for live cell and fixed cell observation	High toxicity	Wang et al. [30]
			Rambold et al. [31]
Fluorescence labeling: Lipi‐probe (Lipi‐Green, LipiDye)	Easy staining	Time consuming than BODIPY staining	Tatenaka et al. [32]
	Low toxicity	Weak signal than BODIPY 493/503	Yamaguchi et al. [33]
	Can be used for live cell and fixed cell observation		
Reporter protein (class 2): PLINs	Cytoplasmic LDs can be visualized	Overexpression (microinjection) required	Wilson et al. [34]
	Can be used for live cell and fixed cell observation	Concerned with unexpected effects	Lumaquin et al. [35]
			Beller et al. [36]
Reporter protein (class 1): HPos, LiveDrop	LD formation can be visualized	Overexpression (microinjection) required	Wilfling et al. [7]
	Can be used for live cell and fixed cell observation		Wang et al. [9]
			Kassan et al. [37]
			Ibayashi et al. [38]

## 2 Visualization of LDs in Oocytes and Fertilized Embryos

### 2.1 Electron Microscopy

Transmission electron microscopy (EM) is the most versatile method for imaging of LDs; it has the advantage of observing not only the ultrastructure of LDs but also their subcellular localization with surrounding organelles. Typical EM images of an early embryo (2‐cell embryo) are shown in Figure 1B. Before fertilization, LDs tend to be clustered, whereas after fertilization, they are dispersed throughout the cytoplasm [25]. Such dynamic changes of LDs suggest a change in lipid metabolism upon fertilization. Meanwhile, it should be noted with concern that the ultrastructure of LDs may be affected by the fixation process that is required for EM analysis (see review by Ohsaki et al.) [26].

### 2.2 Raman Scattering Microscopy

In recent years, a non‐destructive method for observing intracellular LDs using Raman scattering has been reported [39, 40]. Raman scattering uses spectroscopy to detect the scattered light generated by irradiating molecules with a spectrum [41]. Using Raman scattering microscopy, Bradley et al. successfully observed the morphological changes of LD in mouse oocytes and fertilized embryos [27]. However, given that lipid membranes of organelles with lipid‐rich LDs are highly detected by Raman scattering microscopy, probably because long nonpolar acyl groups in lipids give strong Raman scattering [28], caution must be exercised in terms of specificity. In contrast, because Raman spectra are different for each lipid species, Raman scattering microscopy offers the advantage of live imaging of various lipid species distributed within cells.

### 2.3 Fluorescent Labeling

Intracellular LDs can be labeled easily with neutral lipid fluorescent dyes such as dipyrromethene boron difluoride (BODIPY) 493/503 (commercially available from Thermo Fisher Scientific) or Lipi probes, such as Lipi‐Green and LipiDye (commercially available from Dojindo and Funakoshi, respectively) [32, 33], which pass through the cell membrane and accumulate within LDs. These dyes can be used on a variety of cell types and have the advantage of being applicable to fixed cells and tissue sections in addition to living cells. However, it should be kept in mind that they have the disadvantage of being subject to bleaching during observation.

As with cultured cells, oocytes and fertilized embryos can be cultured with BODIPY 493/503 and Lipi probe for fluorescent imaging of LDs in vitro (Figure 1C) [42, 43]. The incubation time varies considerably for individual reagents. For example, 30 min or less is sufficient for fluorescent observation of LDs using BODIPY 493/503, whereas Lipi probe requires several hours or more. In contrast, Lipi probe is far less damaging to embryonic development than BODIPY 493/503. Specifically, embryonic development is arrested in medium containing BODIPY 493/503, depending on the concentration used, while medium containing Lipi probe has little effect on embryonic development (i.e., live imaging over a period of days) is possible (S.T., personal observation). Thus, it is important to understand that different neutral lipid dyes have advantages and disadvantages for fluorescent labeling of LDs.

Fatty acid derivatives such as BODIPY 558/568 C12 (C12‐Red), in which the fluorophore, BODIPY, is appended to the ω‐terminus of lauric acid, are rapidly incorporated into LDs and are therefore an effective way for visualizing LDs in living cells [4, 29, 30, 37]. Fluorescent observation of oocytes co‐cultured with C12‐Red over time shows that C12‐Red accumulates in LDs (Figure 1C: typical images showing the LDs of oocytes co‐labeled with BODIPY 493/503 and C12‐Red, respectively). However, these fatty acid derivative‐conjugated fluorophores are not as specific as BODIPY 493/503 and Lipi probe because they are also incorporated into organelles such as mitochondria and the Golgi apparatus; however, it should be noted that these properties are useful for investigating fatty acid trafficking and metabolism within cells [31].

### 2.4 Utilization of LD Surface Proteins as Markers

Fluorescent imaging of LDs can also be achieved by the intracellular expression of fusion proteins of LD‐binding proteins and fluorescent proteins. In this regard, reporter proteins comprising fluorescent proteins fused to PLINs (FP‐PLINs) are the most versatile. At the living organism level, some studies have investigated the distribution of LDs in tissues using *Drosophila* and zebrafish in which FP‐PLINs are expressed [34, 35, 36]. However, as mentioned above, PLINs are a class II protein (i.e., a protein specific for growing LDs after budding from the ER), and thus early LDs themselves may not be marked. It is also known that the specificity of PLINs for LDs differs; PLIN1 prefers CE‐abundant LDs, while PLIN2 prefers TG‐abundant LDs [44]. In addition, because PLINs regulate the quantity of LDs, their overexpression may cause unanticipated effects.

To visualize LD biogenesis, a class I LD protein with a minimal amino acid sequence that does not affect lipid metabolism is desirable. HPos protein consists of the N‐terminal sequence of ALDI (33 amino acids including the hydrophobic‐positive sequence) required for ER localization and the C‐terminal sequence (20 amino acids) of caveolin 1, for a total of 53 amino acids [37]. Therefore, the expression of a fusion protein consisting of HPos and a fluorescent protein (such as mCherry, described below) in a cell and embryo can be used for fluorescent imaging of the biosynthesis of all LDs (Figure 1D shows a typical image of MII‐oocyte overexpressing mCherry‐HPos), including initial LDs synthesized in the ER. Similar to HPos, LiveDrop, which consists of the 56‐amino‐acid membrane hairpin domain of GPAT4 fused to a fluorescent protein, has also proven to be effective and binds to LDs faster than the full‐length protein [7, 9].

We recently developed a reporter mouse expressing the mCherry‐HPos systemically [38]. As mentioned above, mCherry‐HPos can mark early LDs synthesized on the ER as well as mature LDs distributed in the cytoplasm. Therefore, mCherry‐HPos mice are valuable reporter mice for understanding the impact of physiological changes, such as obesity and aging, on LD biogenesis in vivo.

## 3 Methods to Investigate the Role of LDs in Oocytes and Fertilized Embryos

While the previous sections have focused primarily on methods for visualizing intracellular LDs, this section approaches the analysis of LDs stored in oocytes and fertilized embryos.

### 3.1 Inhibition of Lipolysis and Lipophagy After Fertilization

A simple way to examine LD metabolism in oocytes and fertilized embryos is to evaluate whether inhibiting lipolysis causes the accumulation of LDs. For example, co‐culturing fertilized embryos with lipase inhibitors such as diethylumbelliferyl phosphate and Atglistatin results in LD accumulation [42], indicating that the catabolism of LDs via lipolysis occurs during early embryonic development. In contrast, when fertilized embryos are co‐cultured with autophagy inhibitors such as wortmannin and bafilomycin A1, embryonic development is arrested [45, 46]. It should be kept in mind that this inhibits the bulk degradation of cytoplasmic components including LDs; however, it should also be noted that it does not inhibit lipophagy alone.

### 3.2 Selective Degradation of LDs by Autophagy

Autophagy is a pathway in which cytoplasmic components sequestered by autophagosomes are delivered to lysosomes for degradation [47]. In general, autophagy is a bulk degradation system, but it can also selectively degrade specific organelles [48, 49]. In such selective autophagy, a substrate, also known as an autophagy adaptor, binds to the target organelle through LC3 on the autophagosome sequesters the target organelle for degradation (see reviewed by Anding et al.) [50].

**FIGURE 2:**
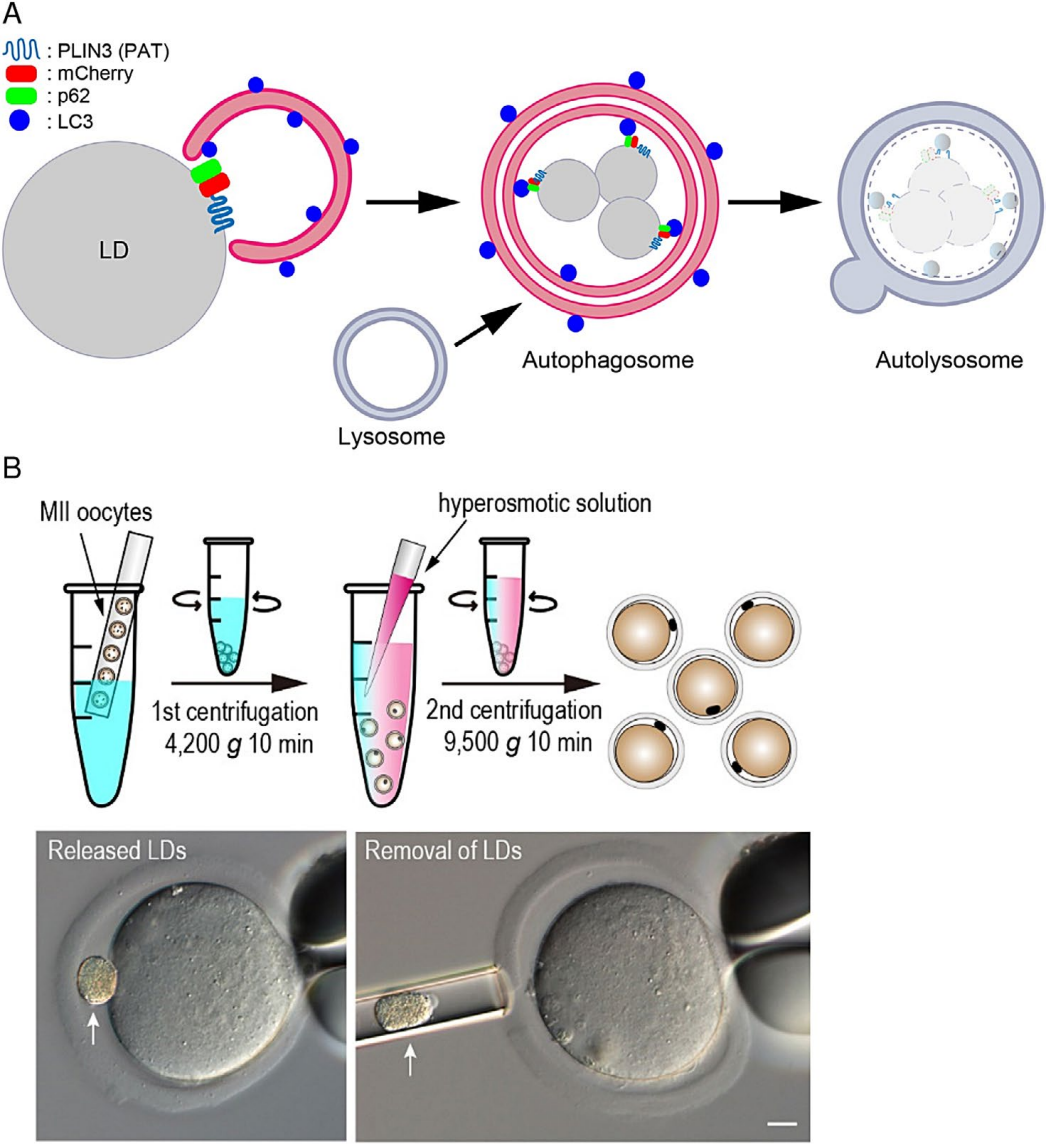
Representative methods to analyze LDs in oocytes and early embryos. (A) Schematic illustration of forced lipophagy. (B) Scheme of LD removal from MII‐oocytes by a two‐step centrifugation. White arrows indicate LDs. Scale bars: 10 μm. See text for details.

Expression of a fusion protein combining a typical autophagy adaptor, Sqstm1/p62 [51], with the PAT domain of PLIN3 (essential for the LD interaction [52]) induces specific localization of p62 to the LD membrane (see Figure 2A). p62 on the LD surface forms a bridge between the LDs and the autophagosomal membrane via interaction with LC3, which stably associates with the membrane. Subsequently, a portion of the LDs is engulfed by the autophagosome and ultimately fuses with lysosomes (forming autolysosomes) for degradation.

By applying this principle to fertilized embryos, specifically, by expressing the autophagy‐adopter p62 on the surface of LDs, autophagy, which actively occurs shortly after fertilization, can degrade p62‐labeled LDs (this method is known as forced lipophagy) [43].

However, because forced lipophagy depends on autophagic activity (i.e., its activity after fertilization can increase and then decrease) [53], not all LDs in fertilized embryos are degraded. Furthermore, given that p62 is a multifunctional signaling molecule, secondary effects of its overexpression must be considered.

### 3.3 Removal of LDs From Mouse MII Oocytes by Two‐Step Centrifugation

Centrifugation of porcine and bovine oocytes and fertilized embryos that contain large amounts of LDs causes LD clustering at the cytoplasmic periphery. In 1997, Nagashima et al. reported that even if LDs are removed from porcine embryos by centrifugation, embryonic development still occurs normally [54]. In 2019, Aizawa et al. successfully removed LDs from mouse MII oocytes with low LD content by combining two‐step centrifugation and hyperosmotic treatment (see Figure 2B): initial low‐speed (first) centrifugation pushes the LDs in the oocytes to the cytoplasmic periphery, followed by high‐speed (second) centrifugation in a hyperosmotic solution, which releases the clustered LDs into the extracellular space (Figure 2B, bottom‐left). Finally, these released LDs were successfully isolated using a fine micropipette (figure 2B, bottom‐right) [42]. The advantage of this method is that the oocytes almost never die after the LDs are removed; therefore, it can be applied to research using oocytes that are completely deficient in LDs (e.g., analysis of fertilization and embryonic development under LD depletion) and research of the removed LDs themselves (e.g., proteomics and lipidomics).

However, this method has several weaknesses. First, it is applicable only to MII oocytes and is not suitable for early embryos after fertilization because the LDs contained in MII oocytes before fertilization are aggregated, depending on the mouse strain used; however, they are dispersed after fertilization, resulting in only the partial release of LDs into the extracellular space. Second, the fertilization ability of MII oocytes after the removal of LDs by this method is reduced, which is thought to be due to zona pellucida hardening by centrifugation and/or hyperosmotic treatment, but intracytoplasmic sperm injection can increase fertilization rates. To apply this method to early embryos after fertilization, individual parameters such as centrifugal speed/temperature and osmotic pressure may need to be optimized further.

## 4 Concluding Remarks

In this review, we have outlined the methods for visualizing and analyzing LDs in oocytes and fertilized embryos, each of which has its own strengths and weaknesses. Hence, it is important to select the appropriate analysis method according to the objectives of the study and to combine independent analysis methods to avoid misinterpretation.

As described here, non‐destructive visualization methods for LDs in oocytes and fertilized embryos have been developed. Such methods may allow us to evaluate embryonic developmental potential by tracing the dynamics of LDs in association with energy metabolism during preimplantation embryonic development. Furthermore, recent studies have shown that dietary lipids are taken up rapidly by oocytes [55]. Raman scattering microscopy may allow us to investigate how the lipid species in oocytes change with diet. The application of such methods to human oocytes and fertilized embryos could contribute to further improving the efficacy of assisted reproductive technologies.

## Funding

This work was supported by Japan Society for the Promotion of Science (23K24489).

## Ethics Statement

*Animal studies*: All mouse experiments were approved and registered by the Animal Care and Use Committee of the National Institutes for Quantum Science and Technology (approval number: 16‐1012‐5).

## Consent

The authors have nothing to report.

## Conflicts of Interest

The authors declare no conflicts of interest.

## Data Availability

The data that support the findings of this study are available from the corresponding author upon reasonable request.
